# Meta-Analysis on Associations of RGS1 and IL12A Polymorphisms with Celiac Disease Risk

**DOI:** 10.3390/ijms17040457

**Published:** 2016-03-30

**Authors:** Cong-Cong Guo, Man Wang, Feng-Di Cao, Wei-Huang Huang, Di Xiao, Xing-Guang Ye, Mei-Ling Ou, Na Zhang, Bao-Huan Zhang, Yang Liu, Guang Yang, Chun-Xia Jing

**Affiliations:** 1 Department of Epidemiology, School of Medicine, Jinan University, Guangzhou 510632, China; guo301@aliyun.com (C.-C.G.); manwang29610@163.com (M.W.); jnuhwh1987@163.com (W.-H.H.); kwstljh@163.com (D.X.); yexingguang1989@sina.com (X.-G.Y.); meiling800569@sina.cn (M.-L.O.); zhangronna@gmail.com (N.Z.); candy2006520@163.com (B.-H.Z.); liuyang199101@126.com (Y.L.); 2 Department of Stomatology of the First Affiliated Hospital of Jinan University, Guangzhou 510632, China; CFDfengdi@163.com; 3 Department of Parasitology, School of Medicine, Jinan University, Guangzhou 510632, China; 4 Key Laboratory of Environmental Exposure and Health in Guangzhou, Jinan University, Guangzhou 510632, China

**Keywords:** celiac disease, IL12A, meta-analysis, polymorphism, RGS1

## Abstract

The pathogenesis of celiac disease (CD) has been related to polymorphisms in the regulator of G-protein signaling 1 (RGS1) and interleukin-12 A (IL12A) genes, but the existing findings are inconsistent. Our aim is to investigate the associations of two single-nucleotide polymorphisms (SNPs) (rs2816316 in RGS1 and rs17810546 in IL12A) with CD risk using meta-analysis. We searched PubMed and Web of Science on RGS1 rs2816316 and IL12A rs17810546 with CD risk. Odds ratio (OR) and 95% confidence interval (CI) of each SNP were estimated. All statistical analyses were performed on Stata 12.0. A total of seven studies were retrieved and analyzed. The available data indicated the minor allele C of rs2816316 was negatively associated with CD (C *vs.* A: OR = 0.77, 95% CI = 0.74–0.80), and a positive association was found for the minor allele G of rs17810546 (G *vs.* A: OR = 1.37, 95% CI = 1.31–1.43). The co-dominant model of genotype effect confirmed the significant associations between *RGS1* rs2816316/*IL12A* rs17810546 and CD. No evidence of publication bias was observed. Our meta-analysis supports the associations of RGS1 and IL12A with CD and strongly calls for further studies to better understand the roles of RGS1 and IL12A in the pathogenesis of CD.

## 1. Introduction

Celiac disease (CD) is a chronic small intestinal immune-mediated enteropathy induced by dietary gluten in genetically predisposed people [1]. Most patients are treated by a life-long gluten-free diet. It has a prevalence of ~1% in many populations worldwide [2]. A genetic linkage study recognized HLA-DQ2 and HLA-DQ8 as the main genetic predisposing factors in the development of CD [3]. However, a genetic-association study by Petronzelli *et al.* [4] identified the effect of HLA contributing to CD was estimated to be only 36%, so non-HLA genes must be considered [5].

Recently, a two-stage genome-wide association study (GWAS) found the contribution of the regions on chromosome 1q31 and 3q25 containing the regulator of G-protein signaling-1 (RGS1) gene and interleukin-12 A (IL12A) gene in the development of CD in European populations [6]. Both of these genes are involved in the T helper cell type 1 (Th1) pathway, which are responsible for mucosal inflammation in active CD [7]. RGS1, a member of the RGS protein family, activated GTPase by attenuating the signaling activity of G-proteins [8]. Moratz *et al.* [9] have reported RGS1 regulates chemokine receptor signaling and participates in B cell activation and proliferation. B cell differentiates into plasma cell and produce anti-tTG/antigliadin antibodies [10]. By interacting with mtTG in the basement membrane region, tTG antibody might lead to enterocyte cytoskeleton and epithelial damage in CD [10]. While IL12A encodes the IL12p35 submit, coincides with IL12p40 to form IL12p70. The heterodimeric IL-12 cytokine has a broad range of bioactivities on T and natural killer cells and it induces the production of interferon-γ (INF-γ) [11]. Activated CD4^+^ T cell produces pro-inflammatory cytokines and results in Th1 response, which is dominantly regulated by INF-γ [12]. Th-1 cytokines promote inflammatory effects and eventually induce enterocyte apoptosis in CD [13].

Several studies have assessed the role for RGS1 rs2816316 (C/A) and IL12A rs17810546 (G/A) in CD development [6,7,14,15,16,17,18]. However, the existing findings were inconsistent even among different ethnic populations in the same study. Thus, we perform a meta-analysis of all available studies to accurately estimate the relationships of RGS1 rs2816316/IL12A rs17810546 polymorphisms with CD risk.

## 2. Results

### 2.1. Eligible Studies and Characteristics

**Figure 1:**
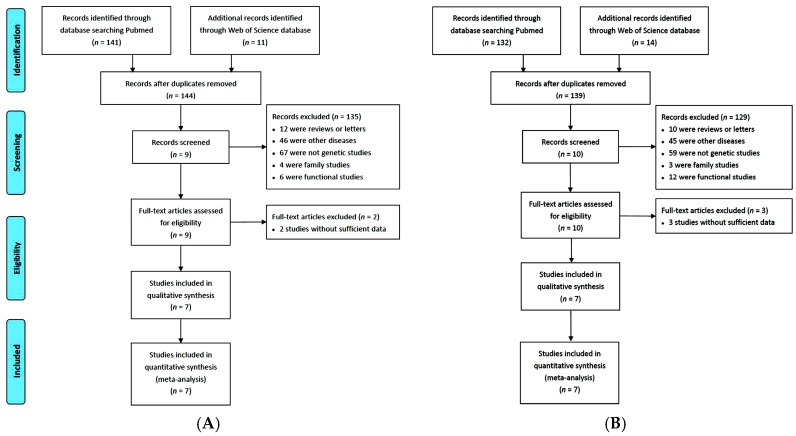
Flow chart showing the literature selection procedure used in this study: (**A**) RGS1 rs2816316; and (**B**) IL12A rs17810546.

According to the search strategy and inclusion criteria, seven studies involving 20 sub-study collections were identified about the associations of RGS1 and IL12A polymorphisms with CD [6,7,14,15,16,17,18], of which 19 were conducted in Europe and one in America (Table 1). The detailed steps of literature search are shown in Figure 1. Both RGS1 and IL12A polymorphisms were studied in 14,936 CD patients and 24,794 controls.

The detailed characteristics of the seven included studies are described in Table 1. Genotype and allele distributions for each study are listed in Table 2 and Table 3. Studies from Hunt *et al.* [6] and Dubois *et al.* [15] included three and twelve independent searches, respectively, which were estimated independently in our meta-analysis. Most of the studies had clear diagnostic criteria and control source, except the Irish Ethnic group by Dubois *et al.* [15] that did not provide details from citation.

### 2.2. Risk of Bias Assessment

The results of bias assessment are presented in Table S1. There are risks of bias in terms of ascertainment of CD (“unclear” in 1 out of 20 studies, 5.0%) and ascertainment of control (“unclear” in 1 out of 20 studies, 5.0%).

### 2.3. Genetic Association between RGS1 rs2816316 and CD Risk

**Figure 2:**
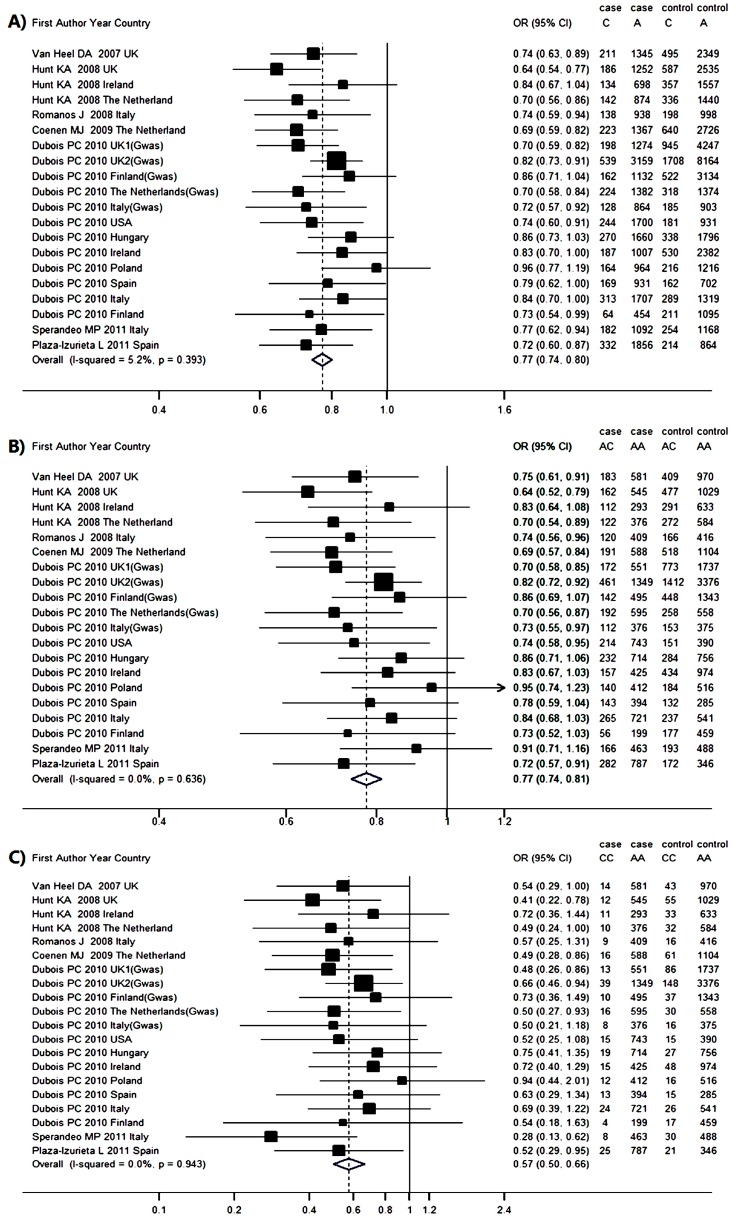
Meta-analysis forest plots of the correlations between RGS1 rs2816316 and celiac disease: (**A**) C *vs.* A; (**B**) AC *vs.* AA; and (**C**) CC *vs.* AA. The vertical dotted line and solid line represent the pooled odds ratio (OR) and the OR equals to 1, respectively. The horizontal solid lines mean the confidence intervals (CI) of OR in each population. And if the upper limit of OR exceed the top scale, arrow would replace the solid line. The size of solid squares corresponds to the weight of the study in the meta-analysis. The length of the hollow diamonds represent the CI of the pooled OR.

**Figure 3:**
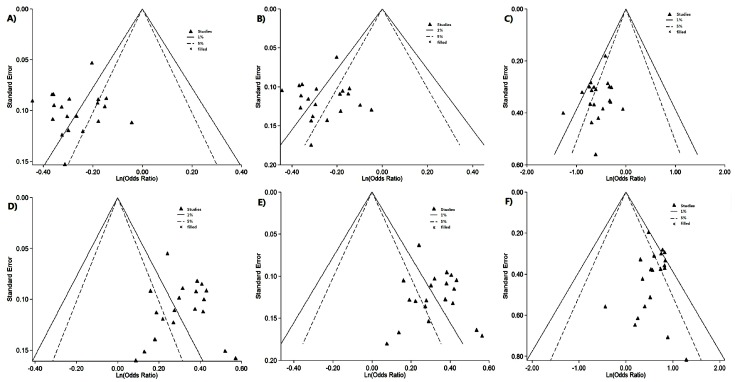
Contour-enhanced funnel plot of IL2/IL21 and SH2B3 genes with CD: (**A**) RGS1 rs2816316 C *vs.* A; (**B**) RGS1 rs2816316 AC *vs.* AA; (**C**) RGS1 rs2816316 CC *vs.* AA; (**D**) IL12A rs17810546 G *vs.* A; (**E**) IL12A rs17810546 AG *vs.* AA; and (**F**) IL12A rs17810546 GG *vs.* AA.

Seven studies reported association between rs2816316 and CD, which involved 14,936 cases and 24,794 controls. Hardy–Weinberg equilibrium (HWE) test showed no study was considered to be disequilibrium. The pooled frequency of the minor allele C was 14.0% (95% CI = 13.6%–14.5%) in the CD groups and 17.5% (95% CI = 16.9%–18.2%) in the control groups. The population-attributable risk (PAR) for allele C was 4.19%. The odds ratio (OR) (C *vs.* A) was estimated by the fix-effect model with less across-study heterogeneity (*p* = 0.393, *I*^2^ = 5.2%), with a pooled OR of 0.77 (95% CI = 0.74–0.80) (Figure 2A). This result suggests that individuals carrying the C allele have a 23% lower risk of developing CD than those carrying the A allele. Harbord (*p* = 0.622) and Peters (*p* = 0.775) tests suggested no existence of publication bias. The results in Figure 3A indicated that the pooled OR (C *vs.* A) were stable without any publication bias. The sensitive analysis was performed to make sure that no individual study was entirely responsible for the pooled results. In Table S2, none of the individual studies affect the final conclusion obviously.

Genotype frequency and estimated OR for each study were presented in Figure 2B,C. The OR_1_ (AC *vs.* AA) (*p* = 0.393, *I*^2^ = 5.2%) and the OR_2_ (CC *vs.* AA) (*p* = 0.943, *I*^2^ = 0.0%) were homogenous. The pooled ORs were estimated by the fix-effects model for OR_1_ (0.77, 95% CI = 0.74–0.81, *p* < 0.001) and OR_2_ (0.57, 95% CI = 0.50–0.66, *p* < 0.001), both of which were significant. Harbord and Peters tests in OR_1_ (*p* = 0.741, 0.910) and OR_2_ (*p* = 0.558, 0.553) as well as contour-enhanced funnel plot (Figure 3B,C) showed no publication bias and stable results. The λ = 0.487 (95% CI = 0.319–0.724) suggested a co-dominant model effect was most likely.

### 2.4. Genetic Association between IL12A rs17810546 and CD Risk

**Figure 4:**
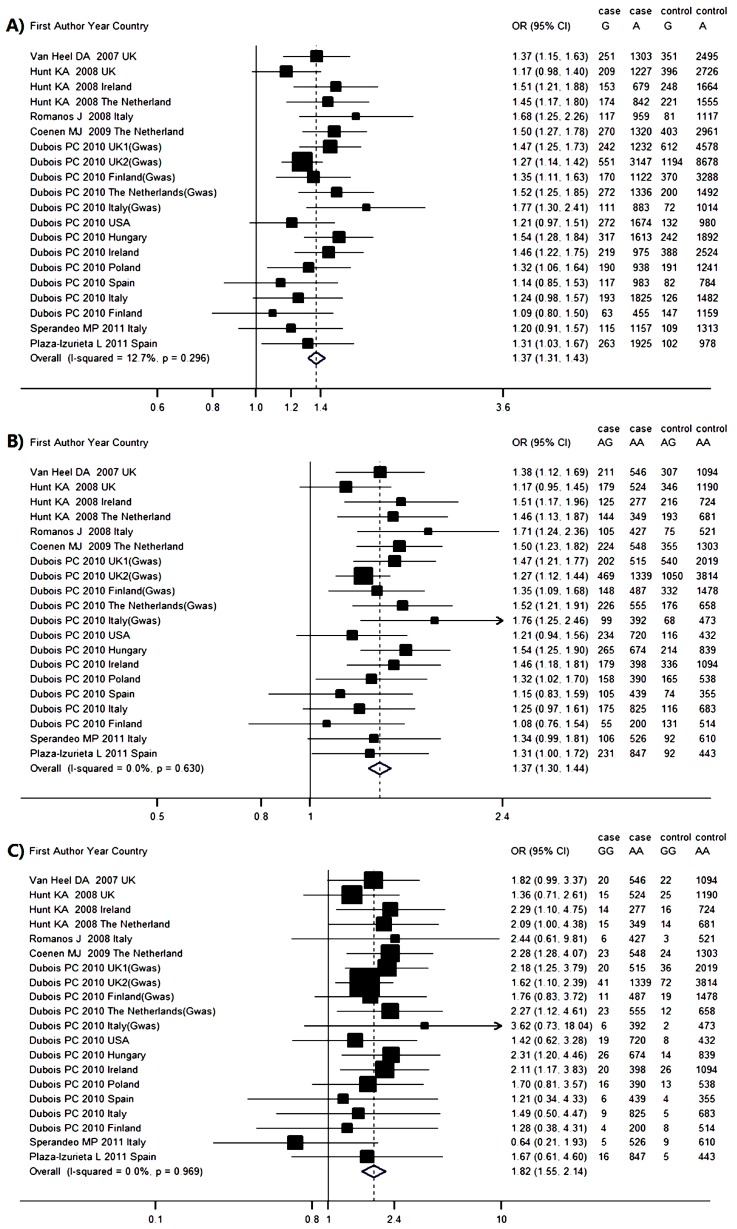
Meta-analysis forest plots of the correlations between IL12A rs17810546 and celiac disease: (**A**) G *vs.* A; (**B**) AG *vs.* AA; and (**C**) GG *vs.* AA. The vertical dotted line and solid line represent the pooled odds ratio (OR) and the OR equals to 1, respectively. The horizontal solid lines mean the confidence intervals (CI) of OR in each population. And if the upper limit of OR exceed the top scale, arrow would replace the solid line. The size of solid squares corresponds to the weight of the study in the meta-analysis. The length of the hollow diamonds represent the CI of the pooled OR.

Seven studies reported association between rs17810546 and CD risk, involving 14,936 cases and 24,794 controls. By using HWE test, most studies were considered to be disequilibrium except for Sperandeo MP *et al.* [17]. The pooled frequency of the minor allele G was 14.3% (95% CI = 13.0%–15.5%) in the CD groups and 10.9% (95% CI = 10.0%–11.7%) in the control groups. The PAR for allele G was 3.87%. The OR (G *vs.* A) was estimated by the fix-effect model with less across-study heterogeneity (*p* = 0.296, and *I*^2^ = 12.7%), with a pooled OR of 1.37 (95% CI = 1.31–1.43) (Figure 4A). This result suggests that individuals carrying the G allele have a 37% higher risk of developing CD than those carrying the A allele. Harbord and Peters tests (*p* = 0.281, 0.281) and contour-enhanced funnel plot (Figure 3D) suggested no publication bias and stable pooled results. The sensitive analysis results in Table S3 indicated none of the individual studies affect the final conclusion obviously.

Genotype frequency and estimated OR for each study were presented in Figure 4B,C. The OR_1_ (AG *vs.* AA) (*p* = 0.630, *I*^2^ = 0.0%) and OR_2_ (GG *vs.* AA) (*p* = 0.969, *I*^2^ = 0.0%) were homogenous. The pooled ORs were estimated by the fix-effects model for OR_1_ (1.37, 95% CI = 1.30–1.44, *p* < 0.001) and OR_2_ (1.82, 95% CI = 1.55–2.14, *p* < 0.001), both of which were significant. Harbord and Peters tests in OR_1_ (*p* = 0.678, 0.642) and OR_2_ (*p* = 0.971, 0.407) and contour-enhanced funnel plot (Figure 3E,F) showed no publication bias and stable results. The λ = 0.562 (95% CI = 0.398–0.816) also suggested a co-dominant model effect was most likely.

## 3. Discussion

In the past few years, several GWAS have identified 40 non-HLA genomic regions harboring 64 CD-associated candidate genes [15]. However, non-HLA SNPs investigated in the present study are not regarded as strong markers of CD. To our knowledge, this is the first meta-analysis to reveal the associations of RGS1 (rs2816316) and IL12A (rs17810546) with CD and represents a pooled total of 14,936 cases and 24,794 controls across different ethnic populations. We found significant evidence for a modest decrease in CD risk associated with the minor C allele in rs2816316, and a modest increase in the minor G allele in rs17810546.

CD is a gluten-sensitive enteropathy and the only adopted treatment is a life-long gluten-free diet [19]. However, the treatment renders ineffective in about 5% of cases whom could develop into enteropathy-associated T cell lymphoma [20]. Thus, there is an urgent need to better understand the pathogenesis of CD, which could propel the development of pharmacologic agents in the treatment of CD. Our study identifies that major allele of rs2816316 and minor allele of rs17810546 are risk factors of CD, which suggests the potential role of RGS1 and IL12A in the treatment of CD. This finding should be confirmed by further biological and clinical studies.

The rs2816316 susceptibility allele is located on chromosome 1q31 and maps 8 kb from distal to the 5′ end of RGS1, which is involved in lymphocyte migration and can influence cell trafficking both in immune system development and exogenous infection [21]. Interestingly, RGS1 has been recently shown to be associated with multiple sclerosis (MS) and type 1 diabetes (T1D), both of which are T cell-mediated diseases [12,22]. The minor allele of rs2816316 is shared with MS and T1D, which suggested that the RGS1 might be an important T cell regulator. Study from Gibbons *et al.* [23] has identified that RGS1 expression is significantly higher in T cells from human gut compared with peripheral blood, especially in intestinal inflammation. Actually, 15%~20% of CD patients suffer from other autoimmune diseases [24], which was strengthened by our study.

rs17810546 locates in a ~70-kb LD block, which is immediately 5′ of IL12A. Similar with the locus RGS1, IL12A has been reported with risk of MS and T1D, confirming the shared genetic factors among different autoimmune diseases [22,25]. In addition, a meta-analysis by Kappen *et al.* [26] identified the genome wide significance association between IL12A rs17810546 and Behcet’s disease, which was also regarded as a Th1 mediated autoimmune disease. The minor allele of rs17810546 showed higher expression level of SCHIP1 gene, located in the same LD block with IL12A gene [7]. IL12A encodes IL12 and induces the Th1 response in the perspective of the etiology of CD [27]. Paajanen *et al.* [28] has shown that the children with CD had the lower expression of IL12A mRNA, which suggested that the major allele of these loci might be a protective factor in CD. In our study, the rs17810546 GG homozygote and heterozygote AG have 82% and 37% increased risk of CD compared with the wide genotype AA, without evidence of between-study heterogeneity, which strengthening the pathogenic effect of IL12A minor allele in CD.

Although several previous studies have investigated the associations between CD risk and RGS1 or IL12A polymorphisms, our meta-analysis is more persuasive and well-documented. First, a total of 20 subsets were included to pool the results for each SNP, dramatically improving the power of statistical analysis. Second, the quality of each included study was desirable, since only studies satisfying the risk-of-bias score test could be included. Third, when we removed the studies one by one to detect the influence of a single study on the pooled OR of other studies, none of the individual studies affect the final conclusion obviously, suggesting the stability of our results. However, this retrospective analysis has several limitations. First, the number of included studies for each SNP is limited (seven studies for rs2816316 and 17810546 each). Nevertheless, this limitation is made up for by the relatively large sample sizes (14,936 cases and 24,794 controls). Second, controls in Italian population are not in Hardy–Weinberg equilibrium [17]. In the sensitivity analysis, however, when this study was excluded, the results of disease associations were not altered. Third, since our study includes almost European ethnicities, the associations among other populations still need to be confirmed.

In summary, our meta-analysis indicates that RGS1 and IL12A are both associated with CD, especially in European populations. The associations should be validated by large-scale, well-designed epidemiological studies in the future.

## 4. Experimental Section

### 4.1. Search Strategy

Studies concerning the associations of RGS1 and IL12A polymorphisms with CD were searched from PubMed and Wed of Science by two reviewers independently. The updating date was 20 August, 2015. The detailed search strategy was as follows: (celiac disease or CD) and (polymorphism or SNP or rs2816316 or rs17810546) or (G-protein signaling-1 or RGS1) or (interleukin-12 A or IL12A). We only considered studies published in English and Chinese. We scanned the titles and abstracts of all relevant articles, manually examined reference lists for additional relevant publications and obtained the full texts of all possibly relevant studies. For multiple articles published with the same subjects, we selected the latest and most complete one.

### 4.2. Inclusion Criteria

Any human population-based association study was included only if it met all three criteria: (1) evaluation of the RGS1 (rs2816316) or IL12A (rs17810546) polymorphism and CD; (2) odds ratio (OR) and its 95% confidence interval (CI) was presented or could be calculated; and (3) there was clear diagnosis of CD. We contacted the authors to acquire indispensable information when their data were incomplete. Studies that did not provide data were excluded.

### 4.3. Data Extraction

Summary data were extracted by two reviewers independently using a standardized form (Table 1). The following information was extracted from each study: (1) name of first author; (2) year of publication; (3) country of population; (4) ethnicity of population; (5) genotyping method; (6) minor allele frequency (MAF) in controls; and (7) sample size of cases and controls. Genotype frequencies of each study and *p*-values for the Hardy–Weinberg equilibrium HWE test in control groups were extracted (Table 2 and Table 3). If there was no detailed genotype data in a study, the theoretical frequency of genotypes was estimated from the MAF of polymorphisms. Any disagreement was resolved through consensus.

### 4.4. Risk of Bias Assessment

The quality of each study was assessed by two reviewers based on a risk-of-bias score for genetic association studies [29]. The score considers 6 domains (Table S1): information bias (ascertainment of outcome and gene), quality control for genotyping, population stratification, confounding bias, selective report of outcomes and HWE assessment in the control group. Each item was classified into “yes”, “no” or “unclear”, which represented low risk, high risk and insufficient information, respectively. Disagreement between the two reviewers was solved by a senior reviewer.

### 4.5. Statistical Analysis

All statistical analyses were performed on Stata 12.0. The association strength of RGS1 rs2816316 and IL12A rs17810546 with CD risk was assessed by OR and its 95%CI. *p* < 0.05 was considered statistically significant in all tests, except for the heterogeneity test in which *p* < 0.10 was used. The HWE of the genotype distribution in a control group was assessed by the *χ*^2^ goodness-of-fit test.

The statistical significance of the pooled OR was determined by the Mantel-Haenszel method. The across-study heterogeneity of allele effects was checked using a Q test and was quantified by *I*^2^ (*I*^2^ < 25%, no; 25%–50%, moderate; 50%–75%, large; >75%, extreme) [30]. If heterogeneity was present (*i.e.*, Q test was significant), the cause of heterogeneity was explored by sensitivity analysis. A random-effect model was used if *I*^2^ > 50%, otherwise, a fixed-effect model was used. The population-attributable risk PAR for mutant allele was calculated based on the results from a discrete-time model [31].

We supposed that A and a were wild-type and mutant alleles, respectively; and AA, Aa and aa were common homozygous, heterozygous, and minor homozygous, respectively. The model-free approach was used to estimate the genotype effect, and two types of ORs for each study: aa *vs.* AA (OR_1_) and Aa *vs.* AA (OR_2_). The model of genetic effect, measured by the parameter lambda (λ: the ratio of logOR_2_ to logOR_1_), was then estimated by the model-free Bayesian approach. The λ represents the heterozygote effect as a proportion of the homozygote variant effect. Furthermore, the λ-value ranges from 0 to 1. We get information about the genetic mode of action as follows: λ = 0 suggests a recessive model (Aa + aa *vs.* AA); λ = 1 suggests a dominant model (AA + Aa *vs.* aa); λ = 0.5 suggests a co-dominant model (AA *vs.* aa; Aa *vs.* aa). λ > 1 or λ < 0 indicates a homozygous or heterosis model, although this is rare. The best genetic model, once identified, is used to collapse the three genotypes into two groups and re-pool the results. WinBugs 1.4.2 was used with vague prior to distributions for parameter estimation (*i.e.*, λ and OR). The models were run with a burn-in of 1000 iterations, followed by 10,000 iterations for parameter estimation.

The Harbord and Peters test were adopted to assess and quantify the publication bias. Additionally, a novel Contour-enhanced meta-analysis funnel plot method was used to combat publication biases [32]. In the sensitive analysis, we removed the studies one by one to identify the influence of a single study on the pooled OR from other studies.

## 5. Conclusions

In conclusion, our study identified RGS1 rs2816316 was negatively associated with CD. In the other hand, IL12A rs17810546 was positively associated with CD. Further studies are required to elucidate how these variants contribute to the susceptibility of CD.

## Figures and Tables

**Table 1:** Characteristics of the included studies.

Author	Year	Country	Ethnicity	Genotyping Method	MAF		Sample Size	
					Control_1	Control_2	Case	Control
Van Heel [18]	2007	UK	European	The Illumina Infinium II assay	0.174	0.123	778	1422
Hunt [6]	2008	UK	European	Infinium assay	0.188	0.127	719	1561
–	–	Ireland	European	GoldenGate assay	0.187	0.130	416	957
–	–	Netherlands	European	GoldenGate assay	0.189	0.124	508	888
Romanos [16]	2009	Italy	European	Taqman probe	0.166	0.067	538	598
Coenen [14]	2009	Netherlands	European	Taqman probe	0.190	0.120	795	1683
Dubois [15]	2010	UK1	European	Illumina Hap300v1-1	0.182	0.118	737	2596
–	–	UK2	European	Illumina 670-QuadCustom_v1	0.173	0.121	1849	4936
–	–	Finland	European	Illumina 670-QuadCustom_v1	0.143	0.101	647	1829
–	–	Netherlands	European	Illumina 670-QuadCustom_v1	0.188	0.118	803	846
–	–	Italy	European	Illumina 670-QuadCustom_v1	0.169	0.067	497	543
–	–	USA	American	Illumina GoldenGate	0.162	0.118	973	555
–	–	Hungary	European	Illumina GoldenGate	0.158	0.113	965	1067
–	–	Ireland	European	Illumina GoldenGate	0.182	0.133	597	1456
–	–	Poland	European	Illumina GoldenGate	0.151	0.133	564	716
–	–	Spain	European	Illumina GoldenGate	0.188	0.095	550	433
–	–	Italy	European	Illumina GoldenGate	0.180	0.078	1010	804
–	–	Finland	European	Illumina GoldenGate	0.162	0.113	259	653
Sperandeo [17]	2011	Italy	European	Taqman probe	0.178	0.077	637	711
Plaza-Izurieta [7]	2011	Spain	European	TaqMan probe	0.199	0.094	1094	540

MAF: minor allele frequency; Control_1: controls for RGS1 rs2816316; Control_2: controls for IL12A rs17810546.

**Table 2:** Genotype frequencies of rs2816316 in celiac disease (CD) and control groups and genotype effects in the meta-analysis.

Author and Year	CD Group			Control Group			C *vs.* A		AC *vs.* AA		CC *vs.* AA		HWE *p* Value
	AA	AC	CC	AA	AC	CC	OR	95% CI	OR	95% CI	OR	95% CI	
Van Heel 2007 UK [18]	581	183	14	970	409	43	0.74	0.63–0.89	0.75	0.61–0.91	0.54	0.29–1.00	0.988
Hunt 2008 UK [6]	545	162	12	1029	477	55	0.64	0.54–0.77	0.64	0.52–0.79	0.41	0.22–0.78	0.976
Hunt 2008 Ireland [6]	293	112	11	633	291	33	0.84	0.67–1.04	0.83	0.64–1.08	0.72	0.36–1.44	0.950
Hunt 2008 Netherlands [6]	376	122	10	584	272	32	0.70	0.56–0.86	0.70	0.54–0.89	0.49	0.24–1.00	0.962
Romanos 2008 Italy [16]	409	120	9	416	166	16	0.74	0.59–0.94	0.74	0.56–0.96	0.57	0.25–1.31	0.908
Coenen 2009 Netherlands [14]	588	191	16	1104	518	61	0.69	0.59–0.94	0.69	0.57–0.84	0.49	0.28–0.86	0.980
Dubois 2010 UK1 [15]	551	172	13	1737	773	86	0.70	0.59–0.82	0.70	0.58–0.85	0.48	0.26–0.86	0.999
Dubois 2010 UK2 [15]	1349	461	39	3376	1412	148	0.82	0.73–0.91	0.82	0.72–0.92	0.66	0.46–0.94	0.980
Dubois 2010 Finland [15]	495	142	10	1343	448	37	0.86	0.71–1.04	0.86	0.69–1.07	0.73	0.36–1.49	0.956
Dubois 2010 Netherlands [15]	595	192	16	558	258	30	0.70	0.58–0.84	0.70	0.56–0.87	0.50	0.27–0.93	0.978
Dubois 2010 Italy [15]	376	112	8	375	153	16	0.72	0.57–0.92	0.73	0.55–0.97	0.50	0.21–1.18	0.934
Dubois 2010 USA [15]	743	214	15	390	151	15	0.74	0.60–1.90	0.74	0.58–0.95	0.52	0.25–1.08	9.933
Dubois 2010 Hungary [15]	714	232	19	756	284	27	0.86	0.73–1.03	0.86	0.71–1.06	0.75	0.41–1.35	0.957
Dubois 2010 Ireland [15]	425	157	15	974	434	48	0.83	0.70–1.00	0.83	0.67–1.03	0.72	0.40–1.29	0.967
Dubois 2010 Poland [15]	412	140	12	516	184	16	0.96	0.77–1.19	0.95	0.74–1.23	0.94	0.44–2.01	0.932
Dubois 2010 Spain [15]	394	143	13	285	132	15	0.79	0.62–1.00	0.78	0.59–1.04	0.63	0.29–1.34	0.952
Dubois 2010 Italy [15]	721	265	24	541	237	26	0.84	0.70–1.00	0.84	0.68–1.03	0.69	0.39–1.22	0.994
Dubois 2010 Finland [15]	199	56	4	459	177	17	0.73	0.54–0.99	0.73	0.52–1.03	0.54	0.18–1.63	0.989
Sperandeo 2011 Italy [17]	463	166	8	488	193	30	0.77	0.62–0.94	0.91	0.71–1.16	0.28	0.13–0.62	0.054
Plaza-Izurieta 2011 Spain [7]	787	282	25	346	172	21	0.72	0.60–0.87	0.72	0.57–0.91	0.52	0.29–0.95	0.947
Overall OR	–	–	–	–	–	–	0.77	0.74–0.80	0.77	0.74–0.81	0.57	0.50–0.67	–

HWE: Hardy–Weinberg equilibrium.

**Table 3:** Genotype frequencies of rs17810546 in CD and control groups and genotype effects in the meta-analysis.

Author and Year	CD Group			Control Group			G *vs.* A		AG *vs.* AA		GG *vs.* AA		HWE *p* Value
	AA	AG	GG	AA	AG	GG	OR	95% CI	OR	95% CI	OR	95% CI	
Van Heel 2007 UK [18]	546	211	20	1094	307	22	1.37	1.15–1.63	1.38	1.12–1.69	1.82	0.99–3.37	0.930
Hunt 2008 UK [6]	524	179	15	1190	346	25	1.17	0.98–1.40	1.17	0.95–1.45	1.36	0.71–2.61	0.979
Hunt 2008 Ireland [6]	277	125	14	724	216	16	1.51	1.21–1.88	1.51	1.17–1.96	2.29	1.10–4.75	0.980
Hunt 2008 Netherlands [6]	349	144	15	681	193	14	1.45	1.17–1.80	1.46	1.13–1.87	2.09	1.00–4.38	0.938
Romanos 2008 Italy [16]	427	105	6	521	75	3	1.68	1.25–2.26	1.71	1.24–2.36	2.44	0.61–9.81	0.865
Coenen 2009 Netherlands [14]	548	224	23	1303	355	24	1.50	1.27–1.78	1.50	1.23–1.82	2.28	1.28–4.07	0.974
Dubois 2010 UK1 [15]	515	202	20	2019	540	36	1.47	1.25–1.85	1.47	1.21–1.77	2.18	1.25–3.79	0.987
Dubois 2010 UK2 [15]	1339	469	41	3814	1050	72	1.27	1.14–1.42	1.27	1.12–1.44	1.62	1.10–2.39	0.977
Dubois 2010 Finland [15]	487	148	11	1478	332	19	1.35	1.11–1.63	1.35	1.09–1.68	1.76	0.83–3.72	0.941
Dubois 2010 Netherlands [15]	555	226	23	658	176	12	1.52	1.25–1.73	1.52	1.21–1.91	2.27	1.12–4.61	0.952
Dubois 2010 Italy [15]	392	99	6	473	68	2	1.77	1.30–2.41	1.76	1.25–2.46	3.62	0.73–18.04	0.788
Dubois 2010 USA [15]	720	234	19	432	116	8	1.21	0.97–1.51	1.21	0.94–1.56	1.42	0.62–3.28	0.946
Dubois 2010 Hungary [15]	674	265	26	839	214	14	1.54	1.28–1.84	1.54	1.25–2.45	2.31	1.20–4.46	0.932
Dubois 2010 Ireland [15]	398	179	20	1094	336	26	1.46	1.22–1.75	1.46	1.18–1.81	2.11	1.17–3.83	0.972
Dubois 2010 Poland [15]	390	158	16	538	165	13	1.32	1.06–1.64	1.32	1.02–1.70	1.70	0.81–3.57	0.932
Dubois 2010 Spain [15]	439	105	6	355	74	4	1.14	0.85–1.53	1.15	0.83–1.59	1.21	0.34–4.33	0.947
Dubois 2010 Italy [15]	825	175	9	683	116	5	1.24	0.98–1.57	1.25	0.97–1.61	1.49	0.50–4.47	0.975
Dubois 2010 Finland [15]	200	55	4	514	131	8	1.09	0.80–1.50	1.08	0.76–1.54	1.28	0.38–4.31	0.914
Sperandeo 2011 Italy [17]	526	106	5	610	92	9	1.20	0.91–1.57	1.34	0.99–1.81	0.64	0.21–1.93	0.029
Plaza-Izurieta 2011 Spain [7]	847	231	16	443	92	5	1.31	1.03–1.67	1.31	1.00–1.72	1.67	0.61–4.60	0.926
Overall OR	–	–	–	–	–	–	1.37	1.31–1.43	1.37	1.30–1.44	1.82	1.55–2.14	–

HWE: Hardy–Weinberg equilibrium.

## Supplementary Materials

Supplementary materials can be found at http://www.mdpi.com/1422-0067/17/4/457/s1.
