# Supplementary material for: Meta-Analysis on Associations of RGS1 and IL12A Polymorphisms with Celiac Disease Risk
Source: Int J Mol Sci. 2016 Mar 30;17(4):457. doi: 10.3390/ijms17040457 (PMC4848913; doi:10.3390/ijms17040457)
Supplement: Supplementary file 1 [file ijms-17-00457-s001.pdf]

Supplementary Materials: Meta-Analysis on Associations of RGS1 and IL12A Polymorphisms with Celiac Disease Risk

Cong-Cong Guo, Man Wang, Feng-Di Cao, Wei-Huang Huang, Di Xiao, Xing-Guang Ye, Mei-Ling Ou, Na Zhang, Bao-Huan Zhang, Yang Liu, Guang Yang and Chun-Xia Jing

Table S1. The risk of bias assessment.

First Author and Year	Ascertainment of Celiac Disease	Ascertainment of Control	Quality Control for Genotyping	Population Stratification	Confounding Bias	Selective Outcome Report	HWE
Van Heel 2007 UK	Yes	Yes	Yes	Yes	Yes	Yes	Yes
Hunt 2008 UK	Yes	Yes	Yes	Yes	Yes	Yes	Yes
Hunt 2008 Ireland	Yes	Yes	Yes	Yes	Yes	Yes	Yes
Hunt 2008 Netherlands	Yes	Yes	Yes	Yes	Yes	Yes	Yes
Romanos 2008 Italy	Yes	Yes	Yes	Yes	Yes	Yes	Yes
Coenen 2009 Netherlands	Yes	Yes	Yes	Yes	Yes	Yes	Yes
Dubois 2010 UK1(GWAS)	Yes	Yes	Yes	Yes	Yes	Yes	Yes
Dubois 2010 UK2(GWAS)	Yes	Yes	Yes	Yes	Yes	Yes	Yes
Dubois 2010 Finland(GWAS)	Yes	Yes	Yes	Yes	Yes	Yes	Yes
Dubois 2010 Netherlands(GWAS)	Yes	Yes	Yes	Yes	Yes	Yes	Yes
Dubois 2010 Italy(GWAS)	Yes	Yes	Yes	Yes	Yes	Yes	Yes
Dubois 2010 USA	Yes	Yes	Yes	Yes	Yes	Yes	Yes
Dubois 2010 Hungary	Yes	Yes	Yes	Yes	Yes	Yes	Yes
Dubois 2010 Ireland	Unclear	Unclear	Yes	Yes	Yes	Yes	Yes
Dubois 2010 Poland	Yes	Yes	Yes	Yes	Yes	Yes	Yes
Dubois 2010 Spain	Yes	Yes	Yes	Yes	Yes	Yes	Yes
Dubois 2010 Italy	Yes	Yes	Yes	Yes	Yes	Yes	Yes
Dubois 2010 Finland	Yes	Yes	Yes	Yes	Yes	Yes	Yes
Sperandeo 2011 Italy	Yes	Yes	Yes	Yes	Yes	Yes	Yes
Plaza-Izurieta 2011 Spain	Yes	Yes	Yes	Yes	Yes	Yes	Yes

HWE: Hardy–Weinberg equilibrium; Yes: low risk of bias; No: high risk of bias; Unclear: unclear risk of bias; GWAS: genome-wide association study.

Table S2. Sensitive analysis for RGS1 rs2816316 (C *vs.* A).

Excluded Study	Pooled OR	95% CI	*p* [a]	I^2 (%)	*p*-Value for I^2
Van Heel 2007 UK	0.77	0.74–0.80	<0.001	9.5	0.340
Hunt 2008 UK	0.78	0.74–0.81	<0.001	0.0	0.606
Hunt 2008 Ireland	0.77	0.73–0.80	<0.001	7.4	0.366
Hunt 2008 Netherlands	0.77	0.74–0.81	<0.001	6.0	0.382
Romanos 2008 Italy	0.77	0.74–0.80	<0.001	9.7	0.337
Coenen 2009 Netherlands	0.77	0.74–0.81	<0.001	2.6	0.425
Dubois 2010 UK1(GWAS)	0.77	0.74–0.81	<0.001	3.4	0.415
Dubois 2010 UK2(GWAS)	0.76	0.73–0.80	<0.001	3.5	0.414
Dubois 2010 Finland(GWAS)	0.76	0.73–0.80	<0.001	3.5	0.413
Dubois 2010 Netherlands(GWAS)	0.77	0.74–0.81	<0.001	5.3	0.392
Dubois 2010 Italy(GWAS)	0.77	0.74–0.80	<0.001	9.0	0.346
Dubois 2010 USA	0.77	0.74–0.80	<0.001	9.4	0.340
Dubois 2010 Hungary	0.76	0.73–0.80	<0.001	1.1	0.442
Dubois 2010 Ireland	0.77	0.73–0.80	<0.001	6.4	0.378
Dubois 2010 Poland	0.76	0.73–0.80	<0.001	0.0	0.586
Dubois 2010 Spain	0.77	0.74–0.80	<0.001	10.0	0.333
Dubois 2010 Italy	0.77	0.73–0.80	<0.001	5.8	0.386
Dubois 2010 Finland	0.77	0.74–0.80	<0.001	9.6	0.337
Sperandeo 2011 Italy	0.77	0.74–0.80	<0.001	10.1	0.331
Plaza-Izurieta 2011 Spain	0.77	0.74–0.80	<0.001	8.1	0.357

[a] *p*: value for Z test; GWAS: genome-wide association study.

Table S3. Sensitive analysis for IL12A rs17810546 (G *vs.* A).

Excluded Study	Pooled OR	95% CI	*p* [a]	I^2 (%)	*p*-Value for I^2
Van Heel 2007 UK	1.37	1.31–1.43	<0.001	17.3	0.242
Hunt 2008 UK	1.38	1.32–1.44	<0.001	4.4	0.403
Hunt 2008 Ireland	1.36	1.30–1.42	<0.001	14.0	0.283
Hunt 2008 Netherlands	1.36	1.30–1.43	<0.001	16.1	0.258
Romanos 2008 Italy	1.36	1.30–1.42	<0.001	9.3	0.342
Coenen 2009 Netherlands	1.36	1.30–1.42	<0.001	12.0	0.308
Dubois 2010 UK1(GWAS)	1.36	1.30–1.42	<0.001	14.0	0.282
Dubois 2010 UK2(GWAS)	1.39	1.32–1.45	<0.001	9.0	0.345
Dubois 2010 Finland(GWAS)	1.37	1.31–1.43	<0.001	17.2	0.243
Dubois 2010 Netherlands(GWAS)	1.36	1.30–1.42	<0.001	12.7	0.299
Dubois 2010 Italy(GWAS)	1.36	1.30–1.42	<0.001	5.5	0.389
Dubois 2010 USA	1.37	1.31–1.44	<0.001	12.2	0.305
Dubois 2010 Hungary	1.36	1.30–1.42	<0.001	10.2	0.330
Dubois 2010 Ireland	1.36	1.30–1.42	<0.001	15.2	0.268
Dubois 2010 Poland	1.37	1.31–1.43	<0.001	16.9	0.248
Dubois 2010 Spain	1.37	1.31–1.44	<0.001	11.2	0.317
Dubois 2010 Italy	1.37	1.31–1.43	<0.001	14.8	0.273
Dubois 2010 Finland	1.37	1.31–1.44	<0.001	8.9	0.346
Sperandeo 2011 Italy	1.37	1.31–1.43	<0.001	13.7	0.287
Plaza-Izurieta 2011 Spain	1.37	1.31–1.43	<0.001	16.9	0.248

[a] *p*: value for Z test; GWAS: genome-wide association study.
